# An Insight Into the Molecular Mechanism of Berberine Towards Multiple Cancer Types Through Systems Pharmacology

**DOI:** 10.3389/fphar.2019.00857

**Published:** 2019-08-06

**Authors:** Pengfei Guo, Chuipu Cai, Xiaoqin Wu, Xiude Fan, Wei Huang, Jingwei Zhou, Qihui Wu, Yujie Huang, Wei Zhao, Fengxue Zhang, Qi Wang, Yongbin Zhang, Jiansong Fang

**Affiliations:** ^1^Science and Technology Innovation Center, Guangzhou University of Chinese Medicine, Guangzhou, China; ^2^Laboratory of Experimental Animal, Guangzhou University of Chinese Medicine, Guangzhou, China; ^3^School of Basic Medical Sciences, Guangzhou University of Chinese Medicine, Guangzhou, China; ^4^Lerner Research Institute, Cleveland Clinic, Cleveland, OH, United States; ^5^Institute of Clinical Pharmacology, Guangzhou University of Chinese Medicine, Guangzhou, China

**Keywords:** berberine, cancer, systems pharmacology, drug–target interactions, significantly mutated genes

## Abstract

Over the past several decades, natural products with poly-pharmacological profiles have demonstrated promise as novel therapeutics for various complex diseases, including cancer. Berberine (PubChem CID: 2353), a soliloquies quaternary alkaloid, has been validated to exert powerful effects in many cancers. However, the underlying molecular mechanism is not yet fully elucidated. In this study, we summarized the molecular effects of berberine against multiple cancers based on current available literatures. Furthermore, a systems pharmacology infrastructure was developed to discover new cancer indications of berberine and explore their molecular mechanisms. Specifically, we incorporated 289 high-quality protein targets of berberine by integrating experimental drug–target interactions (DTIs) extracted from literatures and computationally predicted DTIs inferred by network-based inference approach. Statistical network models were developed for identification of new cancer indications of berberine through integration of DTIs and curated cancer significantly mutated genes (SMGs). High accuracy was yielded for our statistical models. We further discussed three typical cancer indications (hepatocarcinoma, lung adenocarcinoma, and bladder carcinoma) of berberine with new mechanisms of actions (MOAs) based on our systems pharmacology framework. In summary, this study systematically provides a powerful strategy to identify potential anti-cancer effects of berberine with novel mechanisms from a systems pharmacology perspective.

## Introduction

Natural products with diverse chemical scaffolds have been recognized as an invaluable source of candidates in drug discovery and development for multiple complex diseases, including cancer. Berberine, a plant-derived compound isolated from medicinal plants such as Coptis chinensis and Hydrastis canadensis, had a long history of medicinal application in traditional Chinese medicine (Ayati et al., 2017). As one of the main alkaloids, berberine has been reported to exert potentially beneficial effects on many cancer types, including breast cancer (Kim et al., 2008), bladder cancer (Yan et al., 2011), and hepatocarcinoma (Liu et al., 2011; Zhu et al., 2016). For example, berberine had shown significant inhibitory effect on hepatocellular carcinoma cells and could reduce the volume and weight of tumors in an H22 transplanted tumor model in mice (Li et al., 2015).

**Figure 1:**
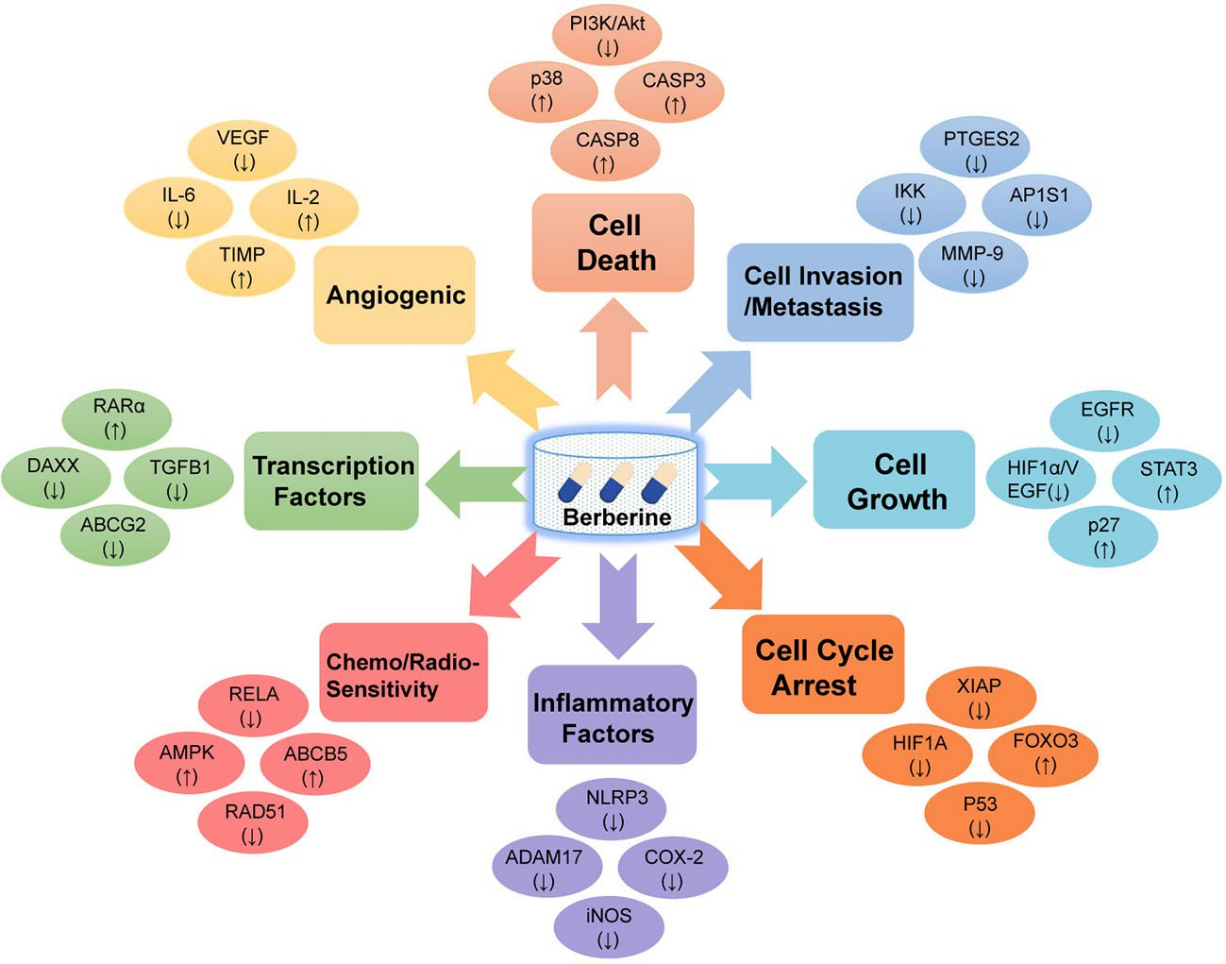
Diagram illustrating the eight potential anti-cancer effects of berberine. Berberine exerts anti-cancer activities *via* targeting various cancer key protein targets, related to cell death, cell invasion and metastasis, cell cycle arrest, cell growth, transcription factors, inflammatory factors, angiogenic, chemo-sensitivity, and radio-sensitivity.

Based on collection of hundreds of berberine-related pharmacological literatures, we systematically summarized eight key mechanisms of anti-cancer effects of berberine, including cell death, cell invasion and metastasis, cell cycle arrest, cell growth, transcription factors, inflammatory factors, angiogenic, chemo-sensitivity, and radio-sensitivity (**Figure 1** and **Supplementary Table S1**). Specifically, apoptosis (programmed cell death) plays a vital role in tumor cell development, differentiation, and proliferation (Ola et al., 2011). Recent study has revealed that berberine could induce apoptosis of human osteosarcoma U2OS cells through inhibiting the PI3K/Akt signaling pathway activation (Chen, 2016). In addition, anti-angiogenesis is a promising strategy for prevention and treatment of multicancer in preclinical or clinical studies in terms of many natural products (Khalid et al., 2016; Kotoku et al., 2016). Previous *in vitro* and *in vivo* experiments have validated that berberine exerted anti-angiogenic effect through inhibiting various proinflammatory and pro-angiogenic factors, including vascular endothelial growth factor (VEGF), interleukin-6 (IL-6), interleukin-2 (IL-2), and metalloproteinase inhibitor (TIMP) (Hamsa and Kuttan, 2012).

Collectively, berberine with polypharmacology has demonstrated its broad anti-cancer properties through targeting various oncogenic pathways and targets. Therefore, systematic exploration of the drug targets of berberine is of great significance for understanding its anti-cancer mechanisms of action (MOAs) and for further excavating its novel cancer indications.

Systems pharmacology-based approaches, as an emerging interdiscipline that combines experimental assays and computational tools, have provided an alternative to understand the therapeutic mechanisms of complex diseases (Fang et al., 2018). Recent studies have demonstrated advanced discovery of new indications for natural products based on systems pharmacology approaches (Fang et al., 2017b; Fang et al., 2019). For example, novel molecular mechanisms of several effective natural products (e.g., resveratrol, quercetin, caffeic acid, and wogonoside) for multiple complex diseases including multi-cancer types and age-related disorders have been identified and validated by various literatures and *in vitro* and *in vivo* experiments (Fang et al., 2017a; Huang et al., 2019). Collectively, systems pharmacology-based approaches have been proved as an effective tool for exploring the poly-pharmacological actions of natural products towards various complex diseases.

**Figure 2:**
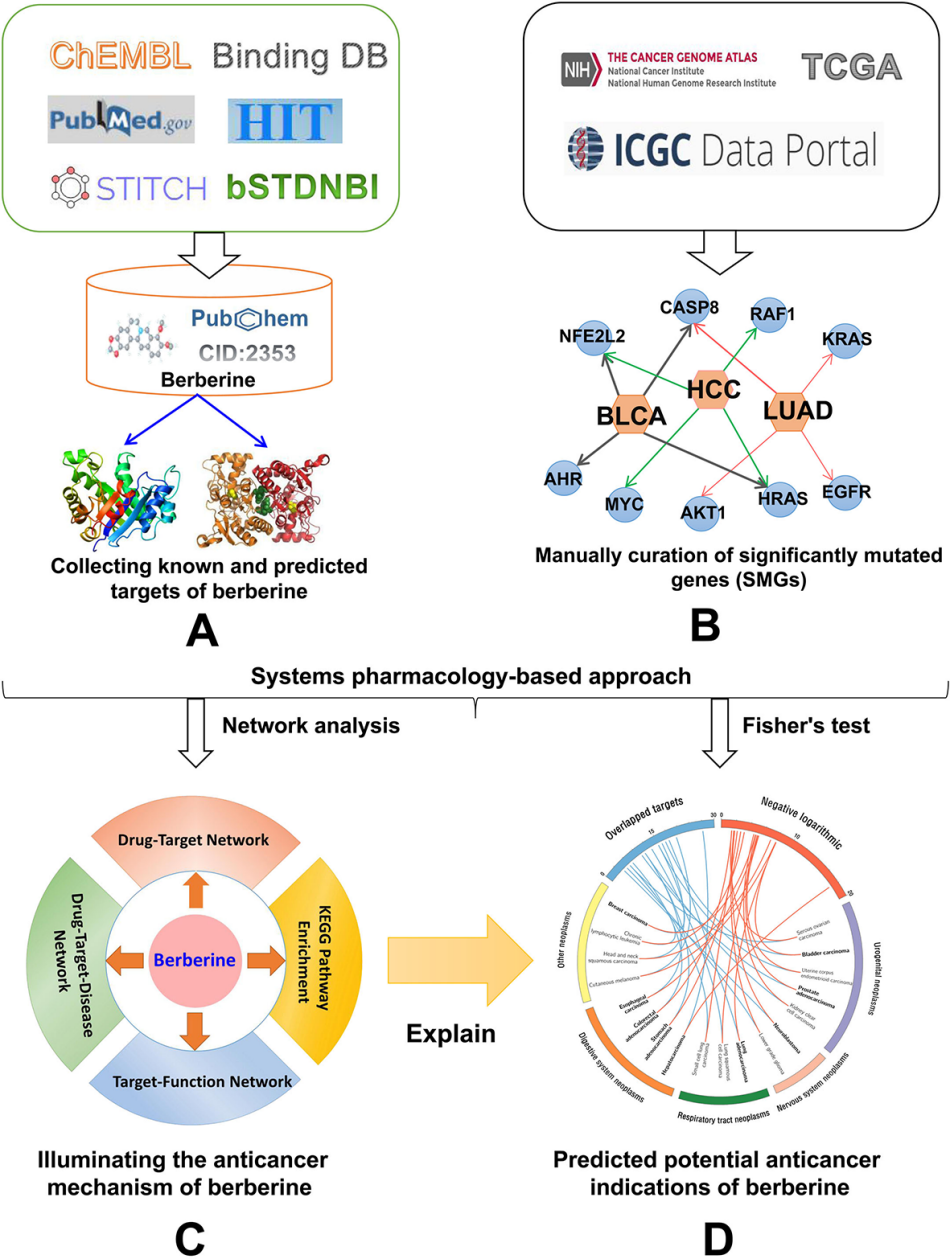
Workflow of a systems pharmacology infrastructure for the identification of cancer indications and exploration of molecular mechanisms of berberine. **(A)** Construction of drug–target network for berberine, **(B)** manual curation of cancer significantly mutated genes (SMGs) for multiple cancer types, **(C)** performing network analyses to explore the anti-cancer mechanism of berberine, and **(D)** statistical network models for prioritizing novel anti-cancer indication of berberine through integrating computationally predicted and known drug targets into the curated cancer SMGs.

In this study, we proposed a systems pharmacology infrastructure to identify new cancer indications of berberine and explore their molecular mechanisms (**Figure 2**). Specifically, we constructed a global DTI network of berberine by integrating both experimentally reported DTIs obtained from literatures and DTIs computationally predicted by our previous predictive network models (Fang et al., 2017c). Besides, a high-quality collection of significantly mutated genes (SMGs) for multiple cancer types was manually collected. On the basis of curated cancer SMGs and DTIs, we built statistical network models with high accuracy to prioritize new cancer indications of berberine and showcased its potential mechanisms. Overall, this study provides a useful systems pharmacology framework to interpret the multi-scale MOAs of berberine in multiple cancer type management, which may give some enlightenment for further treatment of cancer-associated diseases.

## Materials and Methods

### Collection of Known Targets for Berberine

Known targets of berberine were collected by extracting data from four data sources, including HIT (Ye et al., 2011), STITCH (Kuhn et al., 2014), BindingDB (accessed June 2016) (Gilson et al., 2016), and ChEMBL (Bento et al., 2014). For STITCH, we only kept the targets with experimental evidence score higher than 0.7. We totally obtained 66 known targets *via* integrating the four available databases. Besides, we further gathered 238 extra targets of berberine by manually retrieving large-scale pharmacological literatures from PubMed (https://www.ncbi.nlm.nih.gov) with “berberine [title] and cancer” as search terms (**Supplementary Table S2**). After duplicated targets and DTIs were eliminated from non-*Homo sapiens*, 275 high-quality known DTIs were selected for further study (**Supplementary Table S3**).

### Network-Based Target Prediction for Berberine

In a previous study, we have developed statistical network models to predict targets of natural products through a balanced substructure–drug–target network-based inference (bSDTNBI) approach (Fang et al., 2018). The bSDTNBI method utilizes resource-diffusion processes to prioritize potential targets for natural products through integrating known DTI network, drug–substructure linkages, and new input drug–substructure linkages (Wu et al., 2017). For a new input chemical, each of its substructures equally spreads resources to its neighbor nodes layer by layer, and targets obtaining final resources could be regarded as the potential targets of the new chemical. Four parameters (α = β = 0.1, γ = −0.5, and *k* = 2) of bSDTNBI were adopted based on a previous study (Wu et al., 2016). Among them, parameter α was introduced to balance the initial resource allocation of different node types, while β was used to adjust weighted values of different edge types. The third parameter γ was imported to balance the influence of hub nodes in resource-diffusion processes, and the fourth parameter κ denotes the number of resource-diffusion processes. We calculated four substructure items for each compound based on four types of molecular fingerprints from PaDEL-Descriptor (version 2.18) (Yap, 2011), including Substructure (FP4), Klekota-Roth (KR), MACCS, and PubChem. Among the four network models generated with different types of fingerprints, bSDTNBI_KR performed best with the highest values of precision (*P* = 0.049), recall (*R* = 0.752), precision enhancement (Ep = 27.02), recall enhancement (eR = 27.24), and the area under the receiver operating characteristic curve (AUC = 0.959). Finally, the best model built based on KR molecular fingerprint was selected to predict the new targets of berberine. The top 20 predicted candidates were used for further study (**Supplementary Table S3**).

### Significantly Mutated Genes (SMG) for Multiple Cancer Types

We collected 804 SMGs for 28 cancer types/subtypes from a previous study (Cheng et al., 2016), including glioblastoma multiforme (GBM), serous ovarian adenocarcinoma (SOC), stomach adenocarcinoma (STAD), colorectal adenocarcinoma (CRAC), breast carcinoma (BRCA), uterine corpus endometrioid (UCEC), medulloblastoma (MBL), acute myeloid leukemia (AML), cutaneous melanoma (CM), lung squamous cell (SQCC), thyroid carcinoma (THCA), lung adenocarcinoma (LUAD), kidney clear cell (CCSK), head and neck squamous (HNSCC), small cell lung (SCLC), lower grade glioma (LGG), bladder carcinoma (BLCA), esophageal carcinoma (EC), prostate adenocarcinoma (PRAD), hepatocarcinoma (HCC), neuroblastoma (NBL), chronic lymphocytic leukemia (CLL), pancreas adenocarcinoma (PAC), multiple myeloma (MM), acute lymphocytic leukemia (ALL), non-small cell lung (NSCLC), diffuse large B-cell lymphoma (DLBCL), and pilocytic astrocytoma (PA). Considering a lack of statistical power if the number of SMG for specific cancer types is lower than 20, we further excluded ALL, NSCLC, DLBCL, and PA. All SMGs are annotated using gene Entrez ID, chromosome location, and the official gene symbols from the National Center for Biotechnology Information (NCBI) database (Zhe and Huang, 2002). Finally, 24 cancer types/subtypes covering 804 SMGs were selected for further study (**Supplementary Table S4**).

### Prioritizing Cancer Indications of Berberine

In this study, an integrated statistical network model was generated to prioritize cancer indication of berberine based on drug–target network and cancer SMGs (Cheng et al., 2016; Jiang et al., 2018). We assumed that berberine would exert high potential for the treatment of a specific cancer type if its targets tend to be SMGs of this cancer. For each cancer type/subtype, Fisher’s exact test was utilized to calculate the statistical significance of the enrichment of SMGs for each cancer type in target profiles of berberine. The *P*-values were corrected by Benjamini–Hochberg method (Benjamini and Yekutieli, 2001). We set a cutoff adjusted *P*-value threshold (*q*) < 0.05 to define significantly predicted drug–cancer pairs.

### Network Construction

To further explore the multi-scale MOAs of berberine in treating multiple cancer types, three types of networks were constructed by Cytoscape 3.2.1 software (Shannon et al., 2003): 1) drug–target (D-T) network, which presents the relationship between berberine and its targets; 2) target–function (T-F) network, which illustrates the relationship between cancer-related biological processes and SMGs; and 3) drug–target–disease (D-T-D) network, which reflects a global view of the molecular mechanism of berberine against multiple cancer types. After network analysis, the SMGs were further mapped to DAVID database (https://david.ncifcrf.gov/summary.jsp) for extracting the canonical pathways that were highly associated with these targets (Dennis et al., 2003). Finally, circos plot was used to visualize the predicted cancer indications.

## Results and Discussion

### Construction of the Drug–Target (D-T) Network for Berberine

**Figure 3:**
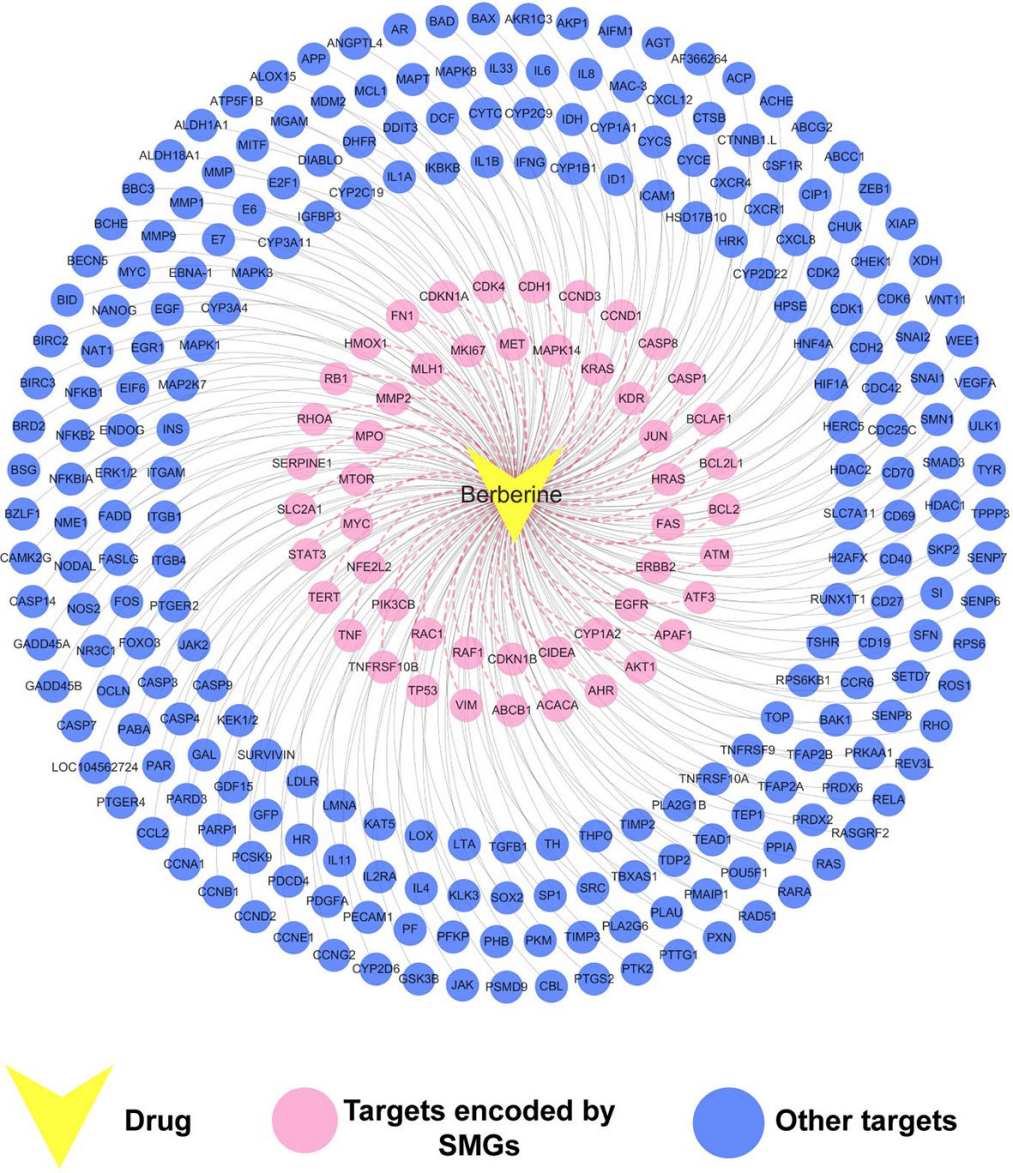
Drug–target (D-T) network of berberine composed of known and predicted targets. The predicted targets were obtained by a balanced substructure–drug–target network-based inference (bSDTNBI) approach. This network includes 289 drug–target interactions connecting berberine and 51 protein targets encoded by significantly mutated genes (SMGs).

The constructed drug–target interaction network (**Figure 3**) of berberine contains 289 interactions, including 275 known targets and 20 predicted targets (**Supplementary Table S3**). *In vitro* and *in vivo* assays in previous studies have validated that five out of the 20 predicted targets could be mediated by berberine, indicating high accuracy of our target prediction approach. These five predicted targets are caspase-3 (CASP3) (Okubo et al., 2017), cellular tumor antigen p53 (TP53) (Qing et al., 2014), caspase-9 (CASP9) (Zhao et al., 2017), nuclear factor NF-kappa-B p105 subunit (NFKB1) (Yu et al., 2014), and mitogen-activated protein kinase 1 (MAPK1) (Song et al., 2015).

We further mapped the 289 protein targets of berberine into the curated cancer SMGs, resulting in 51 cancer-related targets encoded by SMGs (**Supplementary Table S3**). Accumulating evidences indicate that berberine may exert anti-cancer effects through regulating these targets. For instance, signal transducer and activator of transcription 3 (STAT3) are important in various phases of the tumor development, including tumor cell proliferation, survival, invasion, immunosuppression, and inducing and maintaining a pro-carcinogenic inflammatory microenvironment (Fan et al., 2013). A previous study has showed that berberine suppressed tumorigenicity and growth of nasopharyngeal carcinoma (NPC) cells by inhibiting STAT3 activation (Tsang et al., 2013). Recently, a strategy targeting tumor suppressors and apoptosis-related genes provides a rationale for developing more effective approaches and agents for cancer prevention (Sun et al., 2017; López-Cortés et al., 2018; Yamaguchi et al., 2019). Berberine has been observed to activate expression of many tumor apoptosis-related proteins, including caspase-8 (CASP8), tumor necrosis factor-a (TNF-a), and p38 MAPK, and thus induced apoptosis of HeLa cells (Lu et al., 2010). Besides, it has been reported that berberine can decrease expression of mitochondrial-dependent anti-apoptotic factors such as B-cell lymphoma-2 (Bcl-2) and Bcl-2-like protein 1 (BCL2L1) in KB human oral cancer cells (Kim et al., 2015).

Taken together, the observed polypharmacological profiles of berberine motivated us to elucidate its anti-cancer mechanism through systems pharmacology analysis on the interaction between berberine and 51 SMGs.

### Elucidating Molecular Mechanisms of Berberine in Cancer Prevention and Treatment

#### Target–Function Network

**Figure 4:**
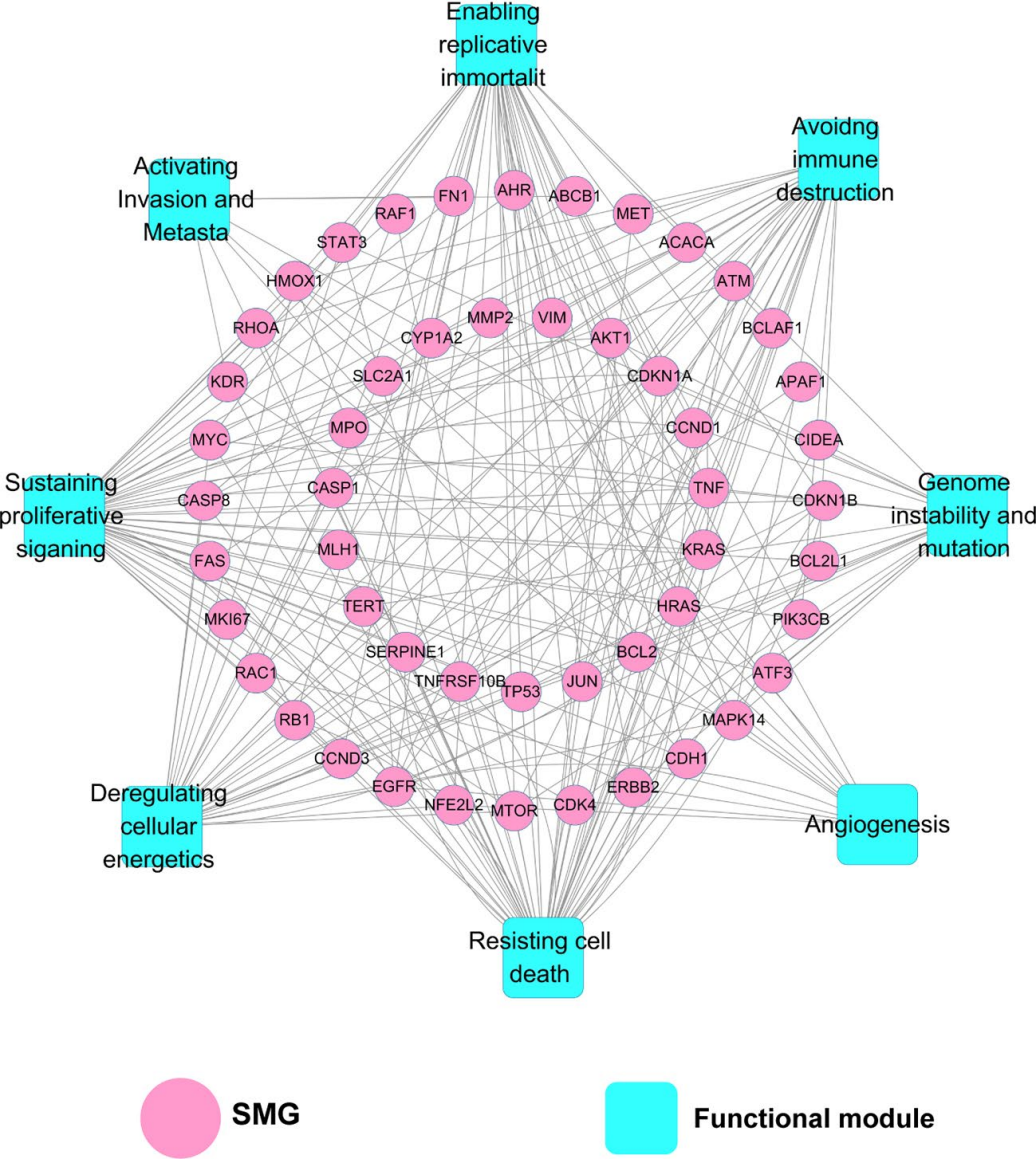
Target–function (T-F) network demonstrating the relationship between cancer-related biological processes and SMGs. A functional module is linked to a target if the target is involved in mechanism of anti-cancer action.

As depicted in **Figure 4**, the target–function (T-F) network is composed of 230 T-F pairs connecting 51 SMG targets and 8 cancer-related functional modules based on the DAVID analysis (**Supplementary Table S5**). The eight functional modules include anti-cancer action associated with sustaining proliferative signaling (Huang et al., 2015), resisting cell death (Chidambara Murthy et al., 2012), deregulating cellular energetics (Tan et al., 2015), enabling replicative immortality (Xiong et al., 2015), avoiding immune destruction (Jiang et al., 2017), genome instability and mutation (Li et al., 2014), angiogenesis (Jie et al., 2011), and activating invasion and metastasis (Tang et al., 2009). On average, each SMG target is involved in six cancer-related functional modules. We found that 25 out of 51 SMG targets are associated with more than five functional modules, indicating the higher potential role of these SMG targets related to cancers. Previous studies of berberine in cancer validated the functional analysis of our T-F network. For instance, berberine could induce cell cycle arrest involved in sustaining proliferative signaling in cholangiocarcinoma KKU-213 and KKU-214 cell lines (Puthdee et al., 2017). Berberine was reported to inhibit metastasis and tumor-induced angiogenesis in human cervical cancer cells as well (Chu et al., 2014).

#### KEGG Enrichment Analysis

In order to further elucidate molecular mechanisms of berberine in cancer prevention and treatment, we performed KEGG pathway enrichment analysis based on the 51 SMGs. After pathways with adjusted *P* (*q*) value higher than 0.05 were excluded, 56 enriched pathways related to cancer pathogenesis were obtained (**Supplementary Table S6**).

Among 56 pathways, PI3K-Akt (hsa04151; *q* = 2.0 × 10^−12^), p53 (hsa04115; *q* = 2.7 × 10^−9^), HIF-1 (hsa04066; *q* = 3.9 × 10^−9^), FoxO (hsa04068; *q* = 4.9 × 10^−9^), VEGF (hsa04370; *q* = 5.7 × 10^−7^), MAPK (hsa04010; *q* = 2.5 × 10^−6^), Ras (hsa04014; *q* = 6.4 × 10^−6^), Jak-STAT (hsa04630; *q* = 9.9 × 10^−4^), mTOR (hsa04150; *q*= 1.5 × 10^−2^), AMPK (hsa04152; *q* = 1.9 × 10^−2^), and NF-kappa B (hsa04064; *q* = 4.0 × 10^−2^) signaling pathways have been confirmed to be associated with berberine in previous literatures (**Table 1**). For example, berberine was reported to inhibit cellular growth and promotes apoptosis by down-regulating PI3K/Akt signaling pathway in breast cancer SKBR-3 cells and hepatoma HepG2 cells (Liu et al., 2011; Kuo et al., 2011). *In vitro* and *in vivo* assays revealed that berberine sensitized drug-resistant breast cancer to doxorubicin (DOX) chemotherapy and directly induced apoptosis through the dose-orchestrated AMPK signaling pathway (Pan et al., 2017). Berberine also induces autophagic cell death through inhibition of mTOR-signaling pathway by suppressing Akt activity and up-regulating P38 MAPK signaling in HepG2 and MHCC97-L cells (Wang et al., 2010). The rest of the 45 enriched pathways prompt the potential anti-cancer acting mechanisms that may be mediated by berberine, which deserve to be validated by experimental assays in the future.

**Table 1:** Summary of the 11 enriched pathways validated to be mediated by berberine in previous literatures.

Pathway ID	Pathway name	Genes	*P* **-value**	PMID
hsa04151	PI3K-Akt signaling pathway	EGFR, HRAS, PIK3CB, MET, TP53, RAF1, BCL2L1, CDK4, KDR, AKT1, CDKN1A, CCND1, KRAS, CDKN1B, CCND3, BCL2, RAC1, MTOR, MYC, FN1	2.03E−12	27081456|25212656
hsa04115	p53 signaling pathway	CDKN1A, CCND1, CCND3, CASP8, SERPINE1, TP53, APAF1, FAS, CDK4, ATM	2.66E−09	20455200
hsa04066	HIF-1 signaling pathway	AKT1, EGFR, HRAS, CCND1, KRAS, PIK3CB, ERBB2, TP53, RAF1, RB1, CDK4	3.89E−09	28775788
hsa04068	FoxO signaling pathway	AKT1, EGFR, HRAS, CCND1, KRAS, PIK3CB, ERBB2, TP53, RAF1, MLH1, CDH1, MYC	4.88E−09	24766860|29360760
hsa04370	VEGF signaling pathway	TNF, MAPK14, BCL2, RAC1, TP53, APAF1, BCL2L1, CASP1	5.72E−07	23869238
hsa04010	MAPK signaling pathway	AKT1, EGFR, HRAS, CCND1, KRAS, PIK3CB, ERBB2, TP53, RAF1, MLH1, CDH1, MYC	2.45E−06	19492307|25212656
hsa04014	Ras signaling pathway	AKT1, EGFR, HRAS, CCND1, KRAS, PIK3CB, ERBB2, TP53, RAF1, RB1, CDK4	6.42E−06	25212656|23159854
hsa04630	Jak-STAT signaling pathway	AKT1, HRAS, KRAS, PIK3CB, JUN, RAC1, RAF1	9.90E−04	26463023
hsa04150	mTOR signaling pathway	TNF, CASP8, APAF1, CASP1	1.50E−02	23159854|20830746
hsa04152	AMPK signaling pathway	EGFR, MAPK14, JUN, RAC1, MET	1.88E−02	28775788
hsa04064	NF-kappa B signaling pathway	TNF, CASP8, APAF1, CASP1	3.97E−02	19107816

#### Drug–Target–Diseases Network

**Figure 5:**
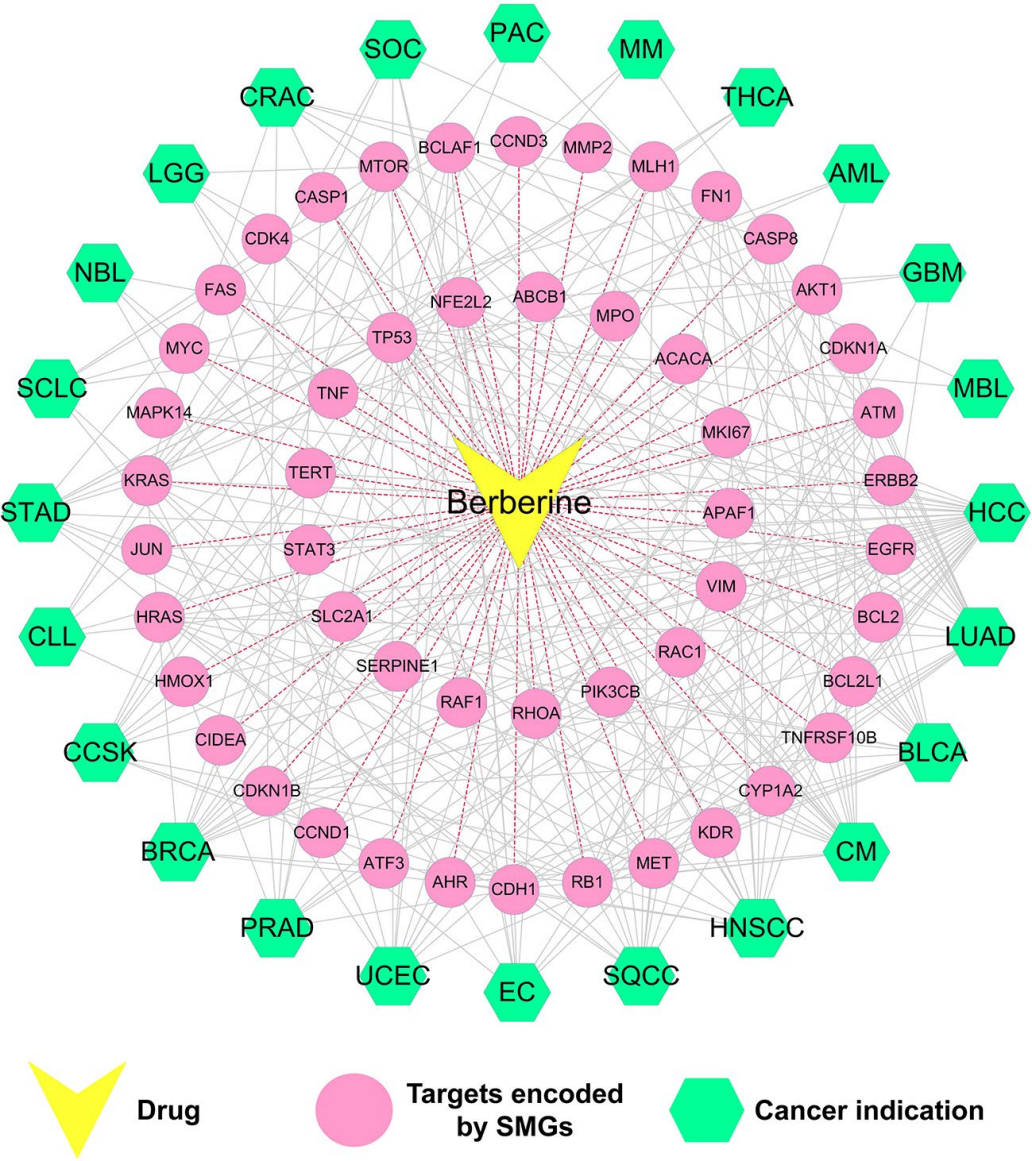
Drug–target–disease (D-T-D) network of berberine. This network shows 51 proteins of berberine encoded by SMGs of 24 types of cancer.

We further built a drug–target–diseases (D-T-D) network *via* mapping 51 SMGs targeted by berberine into multiple cancers. As shown in **Figure 5**, the 51 SMGs are related to 24 types of cancer. On average, each cancer links to nine SMGs, while each SMG is connected to 4.6 cancer types. Network analysis showed that the top 6 SMGs connected to the largest number of cancer types are cellular tumor antigen p53 (TP53), gTPase KRas (KRAS), epidermal growth factor receptor (EGFR), retinoblastoma-associated protein (RB1), serine-protein kinase ATM (ATM), and cadherin-1 (CDH1). Among them, EGFR, a key significantly mutated gene of cancer, is involved in the pathological mechanism of 13 cancer types, including LUAD, HNSCC, SQCC, EC, UCEC, PRAD, BRCA, CCSK, CLL, STAD, LGG, CRAC, and GBM. Previous studies confirmed that berberine can inhibit EGFR signal pathway in several cancer types, including STAD (Wang et al., 2016), PRAD (Huang et al., 2015), and CRAC (Wang et al., 2013). Besides, berberine acts in specific tumor by regulating multiple SMGs. For instance, cellular tumor antigen p53 (TP53) (Wilson et al., 2010), RAC-alpha serine/threonine-protein kinase (AKT1) (López-Cortés et al., 2018), and cyclin-dependent kinase inhibitor 1B (CDKN1B) (Cusan et al., 2018) are highly correlated with breast cancer. Accumulating evidences demonstrated that berberine can inhibit breast cancer by acting on SMGs such as TP53 (Kim et al., 2012; Tan et al., 2015), AKT1 (Kuo et al., 2011), and CDKN1B (Patil et al., 2010).

Briefly, the D-T-D network demonstrated that SMGs targeted by berberine were closely related to multi-cancer types. In the following part, statistical systems pharmacology approach was employed to identify novel cancer indications of berberine and explore the molecular mechanisms.

#### Systems Pharmacology-Based Prediction of Cancer Indications for Berberine

**Figure 6:**
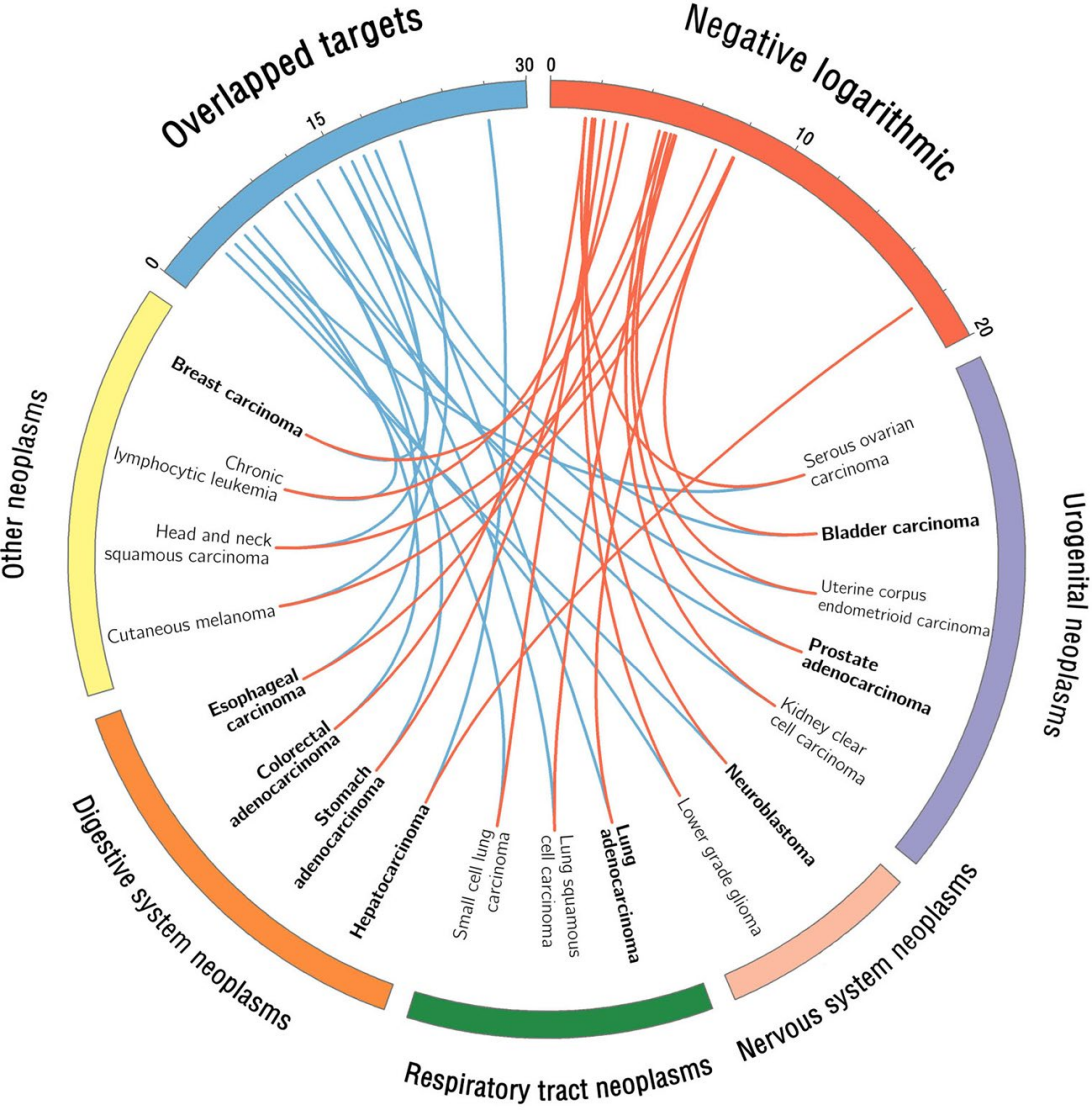
Circos plot visualizes the predicted cancer indications of berberine. The red connected lines represent the calculated −Log_10_ (*q*) value of each berberine-cancer type pair based on Fisher’s exact test, while the blue ones represent the corresponding number of overlapped targets. The predicted cancer indications with literature validation were highlighted in bold font. We classified the 18 predicted cancer indications into four neoplasm categories according to Medical Subject Headings (MeSH) system (https://www.ncbi.nlm.nih.gov/mesh/68009371).

As shown in **Figure 6**, a statistical systems pharmacology framework is proposed to prioritize novel cancer indications of berberine based on Fisher’s exact test analysis. We calculated the therapeutic potential of berberine in 24 cancer indications and obtained 18 cancer indications of which adjusted *P* (*q*) values are lower than 0.05 (*q* < 0.05), including HCC (*q* < 1.0 × 10^−5^; −Log10 (*q*) = 19.25), LUAD (*q* < 1.0 × 10^−5^; −Log10 (*q*) = 9.35), BLCA (*q* < 1.0 × 10^−5^; −Log10 (*q*) = 9.31), CM (*q* < 1.0 × 10^−5^; −Log10 (*q*) = 9.29), HNSCC (*q* < 1.0 × 10^−5^; −Log10 (*q*) = 8.52), SQCC (*q* < 1.0 × 10^−5^; −Log10 (*q*) = 6.74), EC (*q* < 1.0 × 10^−5^; −Log10 (*q*) = 6.66), UCEC (*q* < 1.0 × 10^−5^; −Log10 (*q*) = 6.52), PRAD (*q* = 1.15 × 10^−5^; −Log10 (*q*) = 6.32), BRCA (*q* = 1.33 × 10^−5^; −Log10 (*q*) = 6.26), CCSK (*q* = 2.30 × 10^−5^; −Log10 (*q*) = 6.02), CLL (*q* = 0.55 × 10^−3^; −Log10 (*q*) = 4.64), STAD (*q* = 1.76 × 10^−3^; −Log10 (*q*) = 4.14), SCLC (*q* = 5.33 × 10^−3^; −Log10 (*q*) = 3.65), NBL (*q* = 1.29 × 10^−2^; −Log10 (*q*) = 3.27), LGG (*q* = 1.67 × 10^−2^; −Log10 (*q*) = 3.16), CRAC (*q* = 3.21 × 10^−2^; −Log10 (*q*) = 2.87), and SOC (*q* = 3.36 × 10^−2^; −Log10 (*q*) = 2.85) (**Supplementary Table S7**). As listed in **Table 2**, 10 out of the 18 predicted cancer indications of berberine were validated by reported experimental evidences, including HCC, LUAD, BLCA, EC, PRAD, BRCA, STAD, CRAC, and SOC, indicating the high accuracy of our systems pharmacology-based predictive method (success rate = 55.6%).

**Table 2:** Relevant literature evidences of the 18 predicted cancer indications of berberine.

Cancer type	*P*-value (Fisher test)	Adj-*P*	Negative logarithmic	PMID
HCC	5.63E−20	1.35E−18	17.87	26081696|25496992|24942805
LUAD	4.52E−10	1.08E−08	7.96	24766860|26672764|26503561
BLCA	4.92E−10	1.18E−08	7.93	21545798|23065570|10418949
CM	5.12E−10	1.23E−08	7.91	N/A
HNSCC	3.03E−09	7.27E−08	7.14	26503508
SQCC	1.82E−07	4.37E−06	5.36	N/A
EC	2.18E−07	5.23E−06	5.28	28465635|26667771|21858113
UCEC	3.03E−07	7.27E−06	5.14	N/A
PRAD	4.77E−07	1.15E−05	4.94	16505103|26698234|25572870
BRCA	5.53E−07	1.33E−05	4.88	29143794|29414799|28926092
CCSK	9.58E−07	2.30E−05	4.64	N/A
CLL	2.28E−05	5.47E−04	3.26	N/A
STAD	7.32E−05	1.76E−03	2.76	27142767|25837881|18468407
SCLC	2.22E−04	5.33E−03	2.27	N/A
NBL	5.36E−04	1.29E−02	1.89	27235712|19189664|19096576
LGG	6.95E−04	1.67E−02	1.78	N/A
CRAC	1.34E−03	3.21E−02	1.49	23604974|26463023|25954974
SOV	1.40E−03	3.36E−02	1.47	N/A

Among the 18 cancer indications, CM, HNSCC, SQCC, UCEC, CCSK, CLL, SCLC, NBL, and LGG are the unreported cancer indications of berberine, which deserve further preclinical validation. For example, cutaneous melanoma (CM), one of the most aggressive types of cancer, represents a major problem worldwide due to its high incidence and elevated degree of heterogeneity (Jemal et al., 2010; Coricovac et al., 2018). Based on our predictive model, berberine exerted a high potential for anti-CM, with a significant *q* value [*q* < 1.23 × 10^−8^; −Log10 (*q*) = 9.29]. Therefore, the potential of berberine in the prevention and treatment of CM deserves to be further validated.

#### Case Study: Exploring the MOAs of Berberine on Hepatocarcinoma (HCC), Lung Adenocarcinoma (LUAD), and Bladder Carcinoma (BLCA)

**Figure 7:**
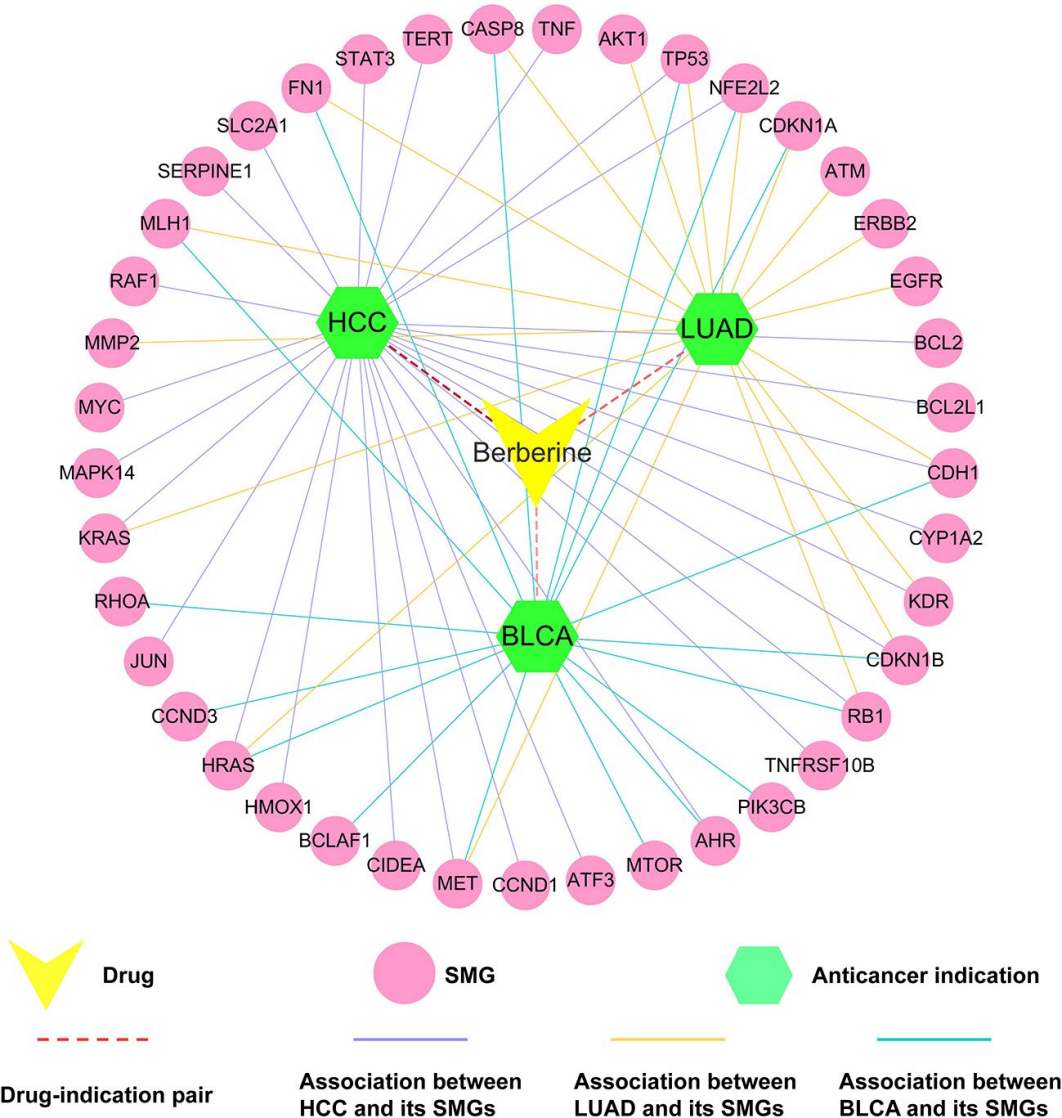
Drug–target–disease (D-T-D) network of berberine on hepatocarcinoma (HCC), lung adenocarcinoma (LUAD), and bladder carcinoma (BLCA). The thickness of the red dotted line represents the predicted association between berberine and three types of tumors.

To further validate the accuracy of statistical network models and predicted anti-cancer targets of berberine, we selected three typical cancer types [HCC (*q* = 5.63 × 10^−20^), LUAD (*q* = 4.52 × 10^−10^), and BLCA (*q* = 4.92 × 10^−10^)] as case studies to illustrate their anti-cancer MOAs (**Figure 7**).

##### Hepatocellular Carcinoma

HCC, the third leading cause of cancer death worldwide, has become one of the most common and prevalent human malignancies in the world (Okubo et al., 2017). *In vitro* assays revealed that berberine can inhibit autophagy in hepatoma cell lines (e.g., HepG2 cells and MHCC97-L cells) by regulating multiple proteins [e.g., mitogen-activated protein kinase 14 (MAPK14), TP53, and phosphatidylinositol 4,5-bisphosphate 3-kinase catalytic subunit beta isoform (PIK3CB)] and pathways (e.g., P38 MAPK signaling), stimulating further development of derivatives for drug-base cancer prevention and treatment (Wang et al., 2010; Liu et al., 2011; Wang et al., 2014). In this study, Fisher’s test showed that berberine played a significant role in treatment of liver cancer (*q* = 5.63 × 10^−20^). In addition, network analysis revealed that berberine bound with 27 HCC-related SMG targets, suggesting its underlying anti-cancer mechanisms of berberine (**Figure 7**). *In vivo* or *in vitro* data have demonstrated that these SMGs are closely relevant to the treatment of cancer by berberine. For example, berberine can inhibit cell proliferation of HepG2, Hep3B, and SNU-182 through up-regulating protein expression of tumor suppressor genes, such as activating transcription factor 3 (ATF3) (Chuang et al., 2017). Furthermore, study revealed that berberine inhibited expression of BCL2, thus reducing autophagic cell death and mitochondrial apoptosis in liver cancer cells, such as HepG2 and MHCC97-L cells (Hur et al., 2010).

##### Lung Adenocarcinoma

LUAD is one of the leading causes of cancer-related death both men and women in the United States. Approximately two million people are diagnosed with lung cancer each year (Torre et al., 2016). Berberine was predicted to have anti-LUAD potential (*q* = 4.52 × 10^−10^). Some previous *in vivo* and *in vitro* studies confirmed our prediction (Mitani et al., 2001; Zheng et al., 2014). Furthermore, berberine is currently being assessed as an anti-LUAD drug in clinical trials (NCT03486496). As shown in **Figure 7**, berberine interacts with 13 LUAD-related SMGs (e.g., matrix metalloproteinase-2), indicating the underlying MOAs of anti-LUAD of berberine. Matrix metalloproteinases (MMPs), one target displayed in our network, is the major protease of LUAD and is associated with tumor invasion and metastasis (Herbst et al., 2000). Study on human lung cancer cell line A549 confirmed that berberine inhibited invasion and growth of tumor cells through decreasing productions of matrix metalloproteinase-2 (MMP2) (Peng et al., 2006).

##### Bladder Carcinoma

BLCA is the most common cancer of the urinary system in the United States (Kaufman et al., 2009). In our network model, berberine is predicted to have a significant relationship with BLCA (*q* = 4.92 × 10^−10^). Meanwhile, our network indicated that berberine interacts with 17 BLCA-related SMGs (e.g., HRAS). According to previous study, the oncogenic ras genes GTPase HRas (HRAS) mutations, endogenously expressed in T24 bladder cancer cell line, were associated with grades and stages of BLCA detected in more than 35% of patients (Buyru et al., 2003). Berberine inhibited cell proliferation and induced cell cycle arrest and apoptosis in BLCA by inhibiting oncogenic H-Ras pathway in BIU-87 and T24 cell lines (Yan et al., 2011).

Taken together, these three case studies against different cancer types (HCC, LUAD, and BLCA) indicate that systems pharmacology approach applied in this study is an effective method for exploring molecular mechanisms of anti-cancer effect of berberine. Meanwhile, the newly predicted tumor types might be promising to further investigate MOAs of berberine.

## Conclusion

Berberine had been observed to exert multiple biological and pharmacological activities with potential benefits to a variety of complex diseases, including cancer. In this study, we proposed an integrated systems pharmacology infrastructure to identify cancer indications of berberine and explore the underlying molecular mechanisms. This work explores the following new anti-cancer characteristics of berberine: i) Through literature mining, we summarize eight mechanisms of anti-cancer effect of berberine; ii) global drug–target network of berberine is constructed by integrating large-scale experimentally reported targets and computationally predicted targets. Mechanisms of action (MOAs) of various anti-cancer effects of berberine are discussed through current drug–target network; iii) a statistical model is developed to prioritize novel cancer indications of berberine through integrating target profiles of berberine and significantly mutated genes in cancer.

Yet several limitations of our approach should be acknowledged. First, although we have integrated a wide range of DTIs from published literatures and publicly available databases, the incompleteness of current drug–target networks may still exist. Recent studies proved that integration of large-scale gene expression profiles of natural products may help to improve the performance of drug–target network model (Yamanishi et al., 2010; Cheng et al., 2012). Second, as it is extremely difficult to obtain information on the active sites of berberine and mutated domain of proteins from public sources, the current study could not explain the MOAs from a microcosmic point of view. Third, experimental assays should be performed to further validate the predicted targets and MOAs of anti-cancer effects of berberine in the future.

In summary, the systems pharmacology framework in this study has provided potential strategies to discover the polypharmacology effects of berberine for the prevention and treatment towards multiple cancers.

## Author Contributions

JF and YZ provided the concept and designed the study. PG and CC conducted the experiments and wrote the manuscript. XW, JZ, XF, QWu, YH, WZ, WH, and FZ participated in the experiments. JF and QWa contributed to revision and proofreading of the manuscript. All authors read and approved the final manuscript.

## Conflict of Interest Statement

The authors declare that the research was conducted in the absence of any commercial or financial relationships that could be construed as a potential conflict of interest.

## Funding

This work was supported by the National Natural Science Foundation of China (Grants 81603318), the youth scientific research training project of GZUCM (2019QNPY05), Research Fund for Characteristic Innovation Projects of Guangdong Province (2016KTSCX013), and Open Tending Project for the Construction of High-Level University (A1-AFD018171Z11027; A1-AFD018171Z11029).

## References

[B1] Ayati S. H. Fazeli B. Momtazi-Borojeni A. A. Afg C. Pirro M. Sahebkar A. (2017). Regulatory effects of berberine on microRNome in cancer and other conditions. Crit. Rev. Oncol. Hematol. 116, 147–158. 10.1016/j.critrevonc.2017.05.008 28693796

[B2] Bento A. P. Gaulton A. Hersey A. Bellis L. J. Chambers J. Davies M. (2014). The ChEMBL bioactivity database: an update. Nucleic Acids Res. 42, 1083–1090. 10.1093/nar/gkt1031 PMC3965067 24214965

[B3] Buyru N. Tigli H. Ozcan F. Dalay N. (2003). Ras oncogene mutations in urine sediments of patients with bladder cancer. J. Biochem. Mol. Biol. 36, 399–402. 10.5483/BMBRep.2003.36.4.399 12895299

[B4] Benjamini Y. Yekutieli D. (2001). The control of the false discovery rate in multiple testing under dependency. Ann. Stat. 29, 1165–1188. 10.1214/aos/1013699998

[B5] Chen Z. Z. (2016). Berberine induced apoptosis of human osteosarcoma cells by inhibiting phosphoinositide 3 kinase/protein kinase B (PI3K/Akt) signal pathway activation. Iran. J. Public Health 45, 578–585. 27398330 PMC4935701

[B6] Cheng F. Liu C. Jiang J. Lu W. Li W. Liu G. (2012). Prediction of drug–target interactions and drug repositioning *via* network-based inference. PLoS Comput. Biol. 8, e1002503. 10.1371/journal.pcbi.1002503 22589709 PMC3349722

[B7] Cheng F. Zhao J. Fooksa M. Zhao Z. (2016). A network-based drug repositioning infrastructure for precision cancer medicine through targeting significantly mutated genes in the human cancer genomes. J. Am. Med. Inform. Assoc. 23, 681–691. 10.1093/jamia/ocw007 27026610 PMC6370253

[B8] Chidambara Murthy K. N. Jayaprakasha G. K. Patil B. S. (2012). The natural alkaloid berberine targets multiple pathways to induce cell death in cultured human colon cancer cells. Eur. J. Pharmacol. 688, 14–21. 10.1016/j.ejphar.2012.05.004 22617025

[B9] Chu S. C. Yu C. C. Hsu L. S. Chen K. S. Su M. Y. Chen P. N. (2014). Berberine reverses epithelial-to-mesenchymal transition and inhibits metastasis and tumor-induced angiogenesis in human cervical cancer cells. Mol. Pharmacol. 86, 609–623. 10.1124/mol.114.094037 25217495

[B10] Chuang T. Y. Wu H. L. Min J. Diamond M. Azziz R. Chen Y. H. (2017). Berberine regulates the protein expression of multiple tumorigenesis-related genes in hepatocellular carcinoma cell lines. Cancer Cell Int. 17, 59. 10.1186/s12935-017-0429-3 28572744 PMC5450260

[B11] Coricovac D. Dehelean C. Moaca E. A. Pinzaru I. Bratu T. Navolan D. (2018). Cutaneous melanoma—a long road from experimental models to clinical outcome: a review. Int. J. Mol. Sci. 19, E1566. 10.3390/ijms19061566 29795011 PMC6032347

[B12] Cusan M. Mungo G. De Marco Zompit M. Segatto I. Belletti B. Baldassarre G. (2018). Landscape of CDKN1B mutations in luminal breast cancer and other hormone-driven human tumors. Front. Endocrinol. 9, 393. 10.3389/fendo.2018.00393 PMC6056726 30065701

[B13] DennisG.ShermanB. T.HosackD. A.YangJ.GaoW.LaneH. C. (2003). DAVID: Database for Annotation, visualization, and Integrated Discovery. Genome Biol. 4, R60. 10.1186/gb-2003-4-9-r60 12734009

[B14] Fan Q. W. Cheng C. K. Gustafson W. C. Charron E. Zipper P. Wong R. A. (2013). EGFR phosphorylates tumor-derived EGFRvIII driving STAT3/5 and progression in glioblastoma. Cancer Cell 24, 438–449. 10.1016/j.ccr.2013.09.004 24135280 PMC3819146

[B15] Fang J. Cai C. Chai Y. Zhou J. Huang Y. Gao L. (2019). Quantitative and systems pharmacology 4. Network-based analysis of drug pleiotropy on coronary artery disease. Eur. J. Med. Chem. 161, 192–204. 10.1016/j.ejmech.2018.10.020 30359818 PMC6263141

[B16] Fang J. Cai C. Wang Q. Lin P. Zhao Z. Cheng F. (2017a). Systems pharmacology-based discovery of natural products for precision oncology through targeting cancer mutated genes. CPT Pharmacometrics Syst. Pharmacol. 6, 177–187. 10.1002/psp4.12172 28294568 PMC5356618

[B17] Fang J. Gao L. Ma H. Wu Q. Wu T. Wu J. (2017b). Quantitative and systems pharmacology 3. Network-based identification of new targets for natural products enables potential uses in aging-associated disorders. Front. Pharmacol. 8, 747. 10.3389/fphar.2017.00747 29093681 PMC5651538

[B18] Fang J. Liu C. Wang Q. Lin P. Cheng F. (2018). In silico polypharmacology of natural products. Brief. Bioinform. 19, 1153–1171. 10.1093/bib/bbx045 28460068

[B19] Fang J. Wu Z. Cai C. Wang Q. Tang Y. Cheng F. (2017c). Quantitative and systems pharmacology. 1. In silico prediction of drug–target interaction of natural products to enable of new targeted cancer therapy. J. Chem. Inf. Model. 57, 2657–2671. 10.1021/acs.jcim.7b00216 28956927 PMC5971208

[B20] Gilson M. K. Liu T. Baitaluk M. Nicola G. Hwang L. Chong J. (2016). BindingDB in 2015: a public database for medicinal chemistry, computational chemistry and systems pharmacology. Nucleic Acids Res. 44, D1045–D1053. 10.1093/nar/gkv1072 26481362 PMC4702793

[B21] Hamsa T. P. Kuttan G. (2012). Antiangiogenic activity of berberine is mediated through the downregulation of hypoxia-inducible factor-1, VEGF, and proinflammatory mediators. Drug Chem. Toxicol. 35, 57–70. 10.3109/01480545.2011.589437 22145808

[B22] Herbst R. S. Yano S. Kuniyasu H. Khuri F. R. Bucana C. D. Guo F. (2000). Differential expression of E-cadherin and type IV collagenase genes predicts outcome in patients with stage I non-small cell lung carcinoma. Clin. Cancer Res. 6, 790–797. 10.1159/000007270 10741698

[B23] Huang Y. Fang J. Lu W. Wang Z. Wang Q. Hou Y. (2019). A systems pharmacology approach uncovers wogonoside as an angiogenesis inhibitor of triple-negative breast cancer by targeting hedgehog signaling. Cell Chem. Biol. 26, 1–16. 10.1016/j.chembiol.2019.05.004 31178408 PMC6697584

[B24] Huang Z. H. Zheng H. F. Wang W. L. Wang Y. Zhong L. F. Wu J. L. (2015). Berberine targets epidermal growth factor receptor signaling to suppress prostate cancer proliferation *in vitro* . Mol. Med. Rep. 11, 2125–2128. 10.3892/mmr.2014.2929 25394789

[B25] Hur J. M. Hyun M. S. Lim S. Y. Lee W. Y. Kim D. (2010). The combination of berberine and irradiation enhances anti-cancer effects *via* activation of p38 MAPK pathway and ROS generation in human hepatoma cells. J. Cell. Biochem. 107, 955–964. 10.1002/jcb.22198 19492307

[B26] Jemal A. Siegel R. Xu J. Ward E. (2010). Cancer statistics, 2010 . CA Cancer J. Clin. 60, 277–300. 10.3322/caac.20073 20610543

[B27] Jiang X. Lu W. Shen X. Wang Q. Lv J. Liu M. (2018). Repurposing sertraline sensitizes non-small cell lung cancer cells to erlotinib by inducing autophagy. JCI Insight 3, 98921. 10.1172/jci.insight.98921 29875309 PMC6124398

[B28] Jiang Y. Huang K. Lin X. Chen Q. Lin S. Feng X. (2017). Berberine attenuates NLRP3 inflammasome activation in macrophages to reduce the secretion of interleukin-1β. Ann. Clin. Lab. Sci. 47, 720–728. 29263046

[B29] Jie S. Li H. Tian Y. Guo D. Zhu J. Gao S. (2011). Berberine inhibits angiogenic potential of Hep G2 cell line through VEGF down-regulation *in vitro* . J. Gastroenterol Hepatol. 26, 179–185. 10.1111/j.1440-1746.2010.06389.x 21175812

[B30] Kaufman D. S. Shipley W. U. Feldman A. S. (2009). Bladder cancer. Lancet 374, 239–249. 10.1016/S0140-6736(09)60491-8 19520422

[B31] Khalid E. B. Ayman E. K. Rahman H. Abdelkarim G. Najda A. (2016). Natural products against cancer angiogenesis. Tumour Biol. 37, 1–24. 10.1007/s13277-016-5364-8 27651162

[B32] Kim J. B. Lee K. M. Ko E. Han W. Lee J. E. Shin I. (2008). Berberine inhibits growth of the breast cancer cell lines MCF-7 and MDA-MB-231. Planta Med. 74, 39–42. 10.1055/s-2007-993779 18203057

[B33] Kim J. S. Oh D. Yim M. J. Park J. J. Kang K. R. Cho I. A. (2015). Berberine induces FasL-related apoptosis through p38 activation in KB human oral cancer cells. Oncol. Rep. 33, 1775–1782. 10.3892/or.2015.3768 25634589 PMC4440222

[B34] Kim S. Han J. Kim N. Y. Lee S. K. Cho D. H. Choi M. Y. (2012). Effect of berberine on p53 expression by TPA in breast cancer cells. Oncol. Rep. 27, 210–215. 10.3892/or.2011.1480 21964832

[B35] Kotoku N. Arai M. Kobayashi M. (2016). Search for anti-angiogenic substances from natural sources. Chem. Pharm. Bull. 64, 128–134. 10.1248/cpb.c15-00744 26833441

[B36] Kuhn M. Szklarczyk D. Pletscher-Frankild S. Blicher T. H. von Mering C. Jensen L. J. (2014). STITCH 4: integration of protein-chemical interactions with user data. Nucleic Acids Res. 42, D401–D407. 10.1093/nar/gkt1207 24293645 PMC3964996

[B37] Kuo H. P. Chuang T. C. Yeh M. H. Hsu S. C. Way T. D. Chen P. Y. (2011). Growth suppression of HER2-overexpressing breast cancer cells by berberine *via* modulation of the HER2/PI3K/Akt signaling pathway. J. Agric. Food Chem. 59, 8216–8224. 10.1021/jf2012584 21699261

[B38] Li J. Li O. Kan M. Zhang M. Shao D. Pan Y. (2015). Berberine induces apoptosis by suppressing the arachidonic acid metabolic pathway in hepatocellular carcinoma. Mol. Med. Rep. 12, 4572–4577. 10.3892/mmr.2015.3926 26081696

[B39] Li Z. Geng Y. N. Jiang J. D. Kong W. J. (2014). Antioxidant and anti-inflammatory activities of berberine in the treatment of diabetes mellitus. Evid. Based Complement. Alternat. Med. 2014, 289264. 10.1155/2014/289264 24669227 PMC3942282

[B40] Liu B. Wang G. Yang J. Pan X. Yang Z. Zang L. (2011). Berberine inhibits human hepatoma cell invasion without cytotoxicity in healthy hepatocytes. PLoS One 6, e21416. 10.1371/journal.pone.0021416 21738655 PMC3123339

[B41] López-Cortés A. Leone P. E. Freire-Paspuel B. Arcos-Villacís N. Guevara-Ramírez P. Rosales F. (2018). Mutational analysis of oncogenic AKT1 gene associated with breast cancer risk in the high altitude ecuadorian mestizo population. Biomed. Res. Int. 2018, 1–10. 10.1155/2018/7463832 PMC6051326 30065942

[B42] Lu B. Hu M. Liu K. Peng J. (2010). Cytotoxicity of berberine on human cervical carcinoma HeLa cells through mitochondria, death receptor and MAPK pathways, and in-silico drug–target prediction. Toxicol. In Vitro 24, 1482–1490. 10.1016/j.tiv.2010.07.017 20656010

[B43] Mitani N. Murakami K. Yamaura T. Ikeda T. Saiki I. (2001). Inhibitory effect of berberine on the mediastinal lymph node metastasis produced by orthotopic implantation of Lewis lung carcinoma. Cancer Lett. 165, 35–42. 10.1016/S0304-3835(00)00710-2 11248416

[B44] Okubo S. Uto T. Goto A. Tanaka H. Nishioku T. Yamada K. (2017). Berberine induces apoptotic cell death *via* activation of caspase-3 and -8 in HL-60 human leukemia cells: nuclear localization and structure–activity relationships. Am. J. Chin. Med. 45, 1497–1511. 10.1142/S0192415X17500811 29025293

[B45] Ola M. S. Nawaz M. Ahsan H. (2011). Role of Bcl-2 family proteins and caspases in the regulation of apoptosis. Mol. Cell Biochem. 351, 41–58. 10.1007/s11010-010-0709-x 21210296

[B46] Pan Y. Zhang F. Zhao Y. Shao D. Zheng X. Chen Y. (2017). Berberine enhances chemosensitivity and induces apoptosis through dose-orchestrated AMPK signaling in breast cancer. J. Cancer 8, 1679–1689. 10.7150/jca.19106 28775788 PMC5535724

[B47] Patil J. B. Kim J. Jayaprakashaa G. K. (2010). Berberine induces apoptosis in breast cancer cells (MCF-7) through mitochondrial-dependent pathway. Eur. J. Pharmacol. 645, 70–78. 10.1016/j.ejphar.2010.07.037 20691179

[B48] Peng P. L. Hsieh Y. S. Wang C. J. Hsu J. L. Chou F. P. (2006). Inhibitory effect of berberine on the invasion of human lung cancer cells *via* decreased productions of urokinase-plasminogen activator and matrix metalloproteinase-2. Toxicol. Appl. Pharmacol. 214, 8–15. 10.1016/j.taap.2005.11.010 16387334

[B49] Puthdee N. Seubwai W. Vaeteewoottacharn K. Boonmars T. Cha’On U. Phoomak C. (2017). Berberine induces cell cycle arrest in cholangiocarcinoma cell lines *via* inhibition of NF-κB and STAT3 pathways. Biol. Pharm. Bull. 40, 751–757. 10.1248/bpb.b16-00428 28566619

[B50] Qing Y. Hu H. Liu Y. Feng T. Meng W. Jiang L. (2014). Berberine induces apoptosis in human multiple myeloma cell line U266 through hypomethylation of p53 promoter. Cell Biol. Int. 38, 563–570. 10.1002/cbin.10206 24843889

[B51] Shannon P. Markiel A. Ozier O. Baliga N. S. Wang J. T. Ramage D. (2003). Cytoscape: a software environment for integrated models of biomolecular interaction networks. Genome Res. 13, 2498–2504. 10.1101/gr.1239303 14597658 PMC403769

[B52] Song Y. C. Lee Y. Kim H. M. Hyun M. Y. Lim Y. Y. Song K. Y. (2015). Berberine regulates melanin synthesis by activating PI3K/AKT, ERK and GSK3β in B16F10 melanoma cells. Int. J. Mol. Med. 35, 1011–1016. 10.3892/ijmm.2015.2113 25716948

[B53] Sun X. Wang S. C. Wei Y. Luo X. Jia Y. Li L. (2017). Arid1a has context-dependent oncogenic and tumor suppressor functions in liver cancer. Cancer Cell 33, 151–152. 10.1016/j.ccell.2017.12.011 PMC5783571 29316428

[B54] Tan W. Li N. Tan R. Zhong Z. Suo Z. Yang X. (2015). Berberine interfered with breast cancer cells metabolism, balancing energy homeostasis. Anticancer Agents Med. Chem. 15, 66–78. 10.2174/1871520614666140910120518 25212656

[B55] Tang F. Wang D. Duan C. Huang D. Wu Y. Chen Y. (2009). Berberine inhibits metastasis of nasopharyngeal carcinoma 5-8F cells by targeting Rho kinase-mediated Ezrin phosphorylation at threonine 567. J. Biol. Chem. 284, 27456–27466. 10.1074/jbc.M109.033795 19651779 PMC2785675

[B56] Tsang C. M. Cheung Y. C. Lui V. W. Yip Y. L. Zhang G. Lin V. W. (2013). Berberine suppresses tumorigenicity and growth of nasopharyngeal carcinoma cells by inhibiting STAT3 activation induced by tumor associated fibroblasts. BMC Cancer 13, 619. 10.1186/1471-2407-13-619 24380387 PMC3890551

[B57] Torre L. A. Siegel R. L. Jemal A. (2016). Lung cancer statistics. Adv. Exp. Med. Biol. 893, 1–19. 10.1007/978-3-319-24223-1 26667336

[B58] Wang J. Yang S. Cai X. Dong J. Chen Z. Wang R. (2016). Berberine inhibits EGFR signaling and enhances the antitumor effects of EGFR inhibitors in gastric cancer. Oncotarget 7, 76076–76086. 10.18632/oncotarget.12589 27738318 PMC5342797

[B59] Wang L. Cao H. Lu N. Liu L. Wang B. Hu T. (2013). Berberine inhibits proliferation and down-regulates epidermal growth factor receptor through activation of Cbl in colon tumor cells. PLoS One 8, e56666. 10.1371/journal.pone.0056666 23457600 PMC3573001

[B60] Wang N. Feng Y. Zhu M. Tsang C. M. Man K. Tong Y. (2010). Berberine induces autophagic cell death and mitochondrial apoptosis in liver cancer cells: the cellular mechanism. J. Cell. Biochem. 111, 1426–1436. 10.1002/jcb.22869 20830746

[B61] Wang N. Zhu M. Wang X. Tan H. Y. Tsao S. W. Feng Y. (2014). Berberine-induced tumor suppressor p53 up-regulation gets involved in the regulatory network of MIR-23a in hepatocellular carcinoma. Biochim. Biophys. Acta. 1839, 849–857. 10.1016/j.bbagrm.2014.05.027 24942805

[B62] Wilson J. R. F. Bateman A. C. Hanson H. An Q. Evans G. Rahman N. (2010). A novel HER2-positive breast cancer phenotype arising from germline TP53 mutations. J. Med. Genet. 47, 771–774. 10.1136/jmg.2010.078113 20805372

[B63] Wu Z. Cheng F. Li J. Li W. Liu G. Tang Y. (2017). SDTNBI: an integrated network and chemoinformatics tool for systematic prediction of drug–target interactions and drug repositioning. Brief. Bioinform. 18, 333–347. 10.1093/bib/bbw012 26944082

[B64] Wu Z. Lu W. Wu D. Luo A. Bian H. Li J. (2016). In silico prediction of chemical mechanism-of-action *via* an improved network-based inference method. Br. J. Pharmacol. 173, 3372–3385. 10.1111/bph.13629 27646592 PMC5738663

[B65] Xiong Y. X. Su H. F. Lv P. Ma Y. Wang S. K. Miao H. (2015). A newly identified berberine derivative induces cancer cell senescence by stabilizing endogenous G-quadruplexes and sparking a DNA damage response at the telomere region. Oncotarget 6, 35625–35635. 10.18632/oncotarget 26462146 PMC4742130

[B66] Yamaguchi R. Lartigue L. Perkins G. (2019). Targeting Mcl-1 and other Bcl-2 family member proteins in cancer therapy. Pharmacol. Ther. 195, 13–20. 10.1016/j.pharmthera.2018.10.009. 30347215

[B67] Yamanishi Y. Kotera M. Kanehisa M. Goto S. (2010). Drug–target interaction prediction from chemical, genomic and pharmacological data in an integrated framework. Bioinformatics 26, i246–i254. 10.1093/bioinformatics/btq176 20529913 PMC2881361

[B68] Yan K. Zhang C. Feng J. Hou L. Yan L. Zhou Z. (2011). Induction of G1 cell cycle arrest and apoptosis by berberine in bladder cancer cells. Eur. J. Pharmacol. 661, 1–7. 10.1016/j.ejphar.2011.04.021 21545798

[B69] Yap C. W. (2011). PaDEL-descriptor: an open source software to calculate molecular descriptors and fingerprints. J. Comput. Chem. 32, 1466–1474. 10.1002/jcc.21707 21425294

[B70] Ye H. Ye L. Kang H. Zhang D. Tao L. Tang K. (2011). HIT: linking herbal active ingredients to targets. Nucleic Acids Res. 39, D1055–D1059. 10.1093/nar/gkq1165 21097881 PMC3013727

[B71] Yu H. Lee H. Herrmann A. Buettner R. Jove R. (2014). Revisiting STAT3 signalling in cancer: new and unexpected biological functions. Nat. Rev. Cancer. 14, 736–746. 10.1038/nrc3818 25342631

[B72] Zhao Y. Jing Z. Lv J. Zhang Z. Lin J. Cao X. (2017). Berberine activates caspase-9/cytochrome *c*-mediated apoptosis to suppress triple-negative breast cancer cells *in vitro* and in *vivo* . Biomed. Pharmacother. 95, 18–24. 10.1016/j.biopha.2017.08.045 28826092

[B73] Zhe W. Huang G. S. (2002). Database resources of the National Center for Biotechnology Information and its application. Chin. Bull. Life Sci. 14, 59–62. 10.1007/BF02943277

[B74] Zheng F. Tang Q. Wu J. J. Zhao S. Y. Liang Z. Y. Li L. (2014). p38α MAPK-mediated induction and interaction of FOXO3a and p53 contribute to the inhibited-growth and induced-apoptosis of human lung adenocarcinoma cells by berberine. J. Exp. Clin. Cancer Res. 33, 36. 10.1186/1756-9966-33-36 24766860 PMC4013801

[B75] Zhu R. X. Seto W. K. Lai C. L. Yuen M. F. (2016). Epidemiology of hepatocellular carcinoma in the Asia-Pacific region. Gut. Liver. 10, 332–339. 10.5009/gnl15257 27114433 PMC4849684

